# Statement of Official Action, with Amounts Contributed for Relief of Physicians, Sufferers by the Late Fire

**Published:** 1871

**Authors:** 


***Light out of Darkness.***

"To every cloud there is a silver lining," and the cloud of gloom which has overshadowed the charred embers of our once beautiful city, is no exception to the rule. Scarcely had the fire fiend arrested his destroying tread, and ere the smoke of the conflagration had subsided; when the tidings of our great calamity which rendered houseless and destitute so many of our professional brethren, in common with their fellow citizens, were flashed across the world, there came back to us, borne upon the wings of the lightning, cheering messages of aid from the members of our noble profession all over the land. The differences of Doctors have been proverbial. They differ now only in a noble emulation to excel each other in generosity to their suffering brothers.

The old proverb, "Three physicians, two atheists," must be altered in this our day, to "Three physicians, two philanthropists." The disaster which has befallen us has given our profession the opportunity to vindicate itself, and to demonstrate on a grand scale that philanthropic spirit which is the real characteristic of the true physician.

Never was there occasion more urgent for philanthropic zeal, and never was occasion more promptly seized, nor zeal more ardently manifested.

On the evening of the 8th of October, the great conflagration began, and continued its ravages until the afternoon of the 9th.

On the 11th, a stated meeting of the New York Pathological Society was held, at which it was—

*Resolved,* That the following communication be sent to the Physicians of Chicago:

*To our Brethren in the Medical Profession of the City of Chicago:*

Believing that in consequence of the recent calamitous fire many of you have suffered serious pecuniary losses, and fearing that some may be in actual want, we desire to be informed what aid we can render you.

We would be glad to inaugurate measures for the establishment of a relief-fund, and to appeal to the medical profession in this city, and throughout the country, in its behalf, provided you confirm its propriety and necessity.

It is perhaps needless to say that, while we would not stay the hand of charity extended to the suffering people of Chicago, without reference to class, this suggestion of relief is not intended for any except those whom we recognize as honorable and legitimate members of our profession.

(Signed) A. L. LOOMIS, M.D., *President.*

G. F. SHRADY, M.D., *Secretary, New York Pathological Society.*

---

A meeting of the members of the medical profession was called in St. Louis, which was called to order by Dr. Gregory, and of which Dr. Marshall was elected Chairman, and Dr. Hurd, Secretary.

Dr. Kinnard offered the following preamble and resolutions, which were adopted:

WHEREAS, many members of our profession, in common with the public at large, have already contributed liberally to the common charitable fund raised by the citizens of St. Louis for the Chicago sufferers; therefore, be it

*Resolved,* That our meeting to-night has been called together to enable the regular medical profession of St. Louis to act in concert in their efforts to aid such worthy medical men of Chicago as may have been rendered destitute by the late disastrous conflagration that laid waste so large a portion of that city.

*Resolved,* That we believe our object can be best accomplished by sending two delegates from the profession of St. Louis to that city, whose business it shall be to diligently inquire into the necessities of our brethren there, and to aid such as may be needy and deserving, as far as practicable, with the amount which may be placed in their hands for that purpose.

*Resolved,* That the committee, on its return, report the prospective needs of the destitute members of the profession in Chicago.

Dr. Hammer moved that a committee of two physicians from each ward be appointed to solicit subscriptions for the object in view.

The motion prevailed, and the following committees were appointed:

Carondelet—Drs. Starkloff and Outen.
First Ward—Drs. Reis and Hickman.
Second Ward—Drs. Fred. Hauck and Castelhuhn.
Third Ward—Drs. Green and Wall.
Fourth Ward—Drs. Porter and Borck.
Fifth Ward—Drs. Hammer and Mudd.
Sixth Ward—Drs. Gill and Bates.
Seventh Ward—Drs. Hill and Newman.
Eighth Ward—Drs. Heyer and Coleman.
Ninth Ward—Drs. Guhman and Hawley.
Tenth Ward—Drs. Baker and Rohlfing.
Eleventh Ward—Drs. Scott and Lingenfelder.
Twelfth Ward—Drs. Carrington and Strotholte.
Elleardville—Dr. Tyler.
Lowell—Dr. Ben Linton.

---

Cheered by these evidences of sympathy from our professional brethren, a large meeting of the members of the medical profession of Chicago was held at No. 797 Wabash Avenue, on Tuesday evening, October 17th, at which Dr. N. S. Davis was elected Chairman, and Dr. E. Andrews Secretary. At which meeting the above communications were read, as also the following telegraphic dispatch:

NEW YORK, Oct. 17, '71.

DR. H. A. JOHNSON,
*Chairman of Sanitary Committee:*

Over two thousand dollars subscribed by medical men this evening for suffering physicians of Chicago. Organize for its distribution, and draw on Dr. S. T. Hubbard, twenty-seven West Ninth st. Further amts. to be reported.

(Signed) E. R. PEASLEE, M.D.,
*Chairman.*

Whereupon Drs. Moses Gunn, E. Andrews, and A. Fisher, having been appointed a committee to recommend suitable persons for a permanent Relief Committee of five, nominated the following gentlemen: Drs. De Laskie Miller, N. S. Davis, T. D. Fitch, Ernst Schmidt, and Walter Hay. Which nomination, having been unanimously confirmed, the following resolutions were adopted:

*Resolved*, That the Committee just chosen is hereby authorized to receive all donations for the relief of respectable physicians, who are sufferers by the late fire, distribute the same at their discretion, and render a strict account with vouchers, to any future meeting, which may be called by the chairman to consider the same.

*Resolved*, That this meeting tender the cordial and heartfelt thanks of the profession of this city, to their brethren in other and distant cities, for the prompt and liberal offers of assistance to the many among us who have lost, by the late terrible fire, not only their homes, clothes, books and instruments, but their practice, and pledge a just use of whatever is given.

On Wednesday, October 18th, the Committee having organized, issued a circular embodying the resolutions passed at the preceding general meeting, together with the following notice:

Contributions may be forwarded at once by Express, or Draft on New York, to Walter Hay, M.D., Secretary Medical Relief Committee, No. 384 Michigan Avenue.

Donations from Publishing Houses, Instrument Makers and Physicians, of Books, Instruments or Apparatus, will be gratefully received; as many of our professional brethren have saved only their lives.

COMMITTEE.
DE LASKIE MILLER, M.D.,
No. 518 Wabash Ave., *Chairman.*
N. S. DAVIS, M.D.,
No. 797 Wabash Ave., *Treasurer.*
ERNST SCHMIDT, M.D.,
No. 387 State Street.
T. D. FITCH, M.D.,
No. 296 West Monroe Street.
WALTER HAY, M.D.,
No. 384 Michigan Ave., *Secretary.*

On the 19th the following telegram was received:

NEW YORK, Oct. 19, '71.

JNO. H. RAUCH, M.D.,

*Board of Health, Chicago:*

Committee meet Saturday night; send full statement.

(Signed) FRANK H. HAMILTON, M.D.

On the 20th Oct., the following notice was published in all the city papers, English and German:

*To Physicians, Sufferers by the Fire:*

The Committee appointed by the physicians of Chicago, to receive and disburse contributions, forwarded by our professional brethren, for the relief of those members of the profession who have suffered by the fire, having organized, respectfully request all regular physicians in good standing, who have sustained losses in this great calamity, to present to the Secretary, or to any member of the Committee, written statements of the amount and character of their respective losses, and of their present necessities (together with former and present addresses) in order that they may be relieved with the least possible delay, and that justice may be done to all in the distribution of this noble charity.

DE LASKIE MILLER, M.D.,
No. 518 Wabash Ave., *Chairman.*
N. S. DAVIS, M.D.,
No. 797 Wabash Ave., *Treasurer.*
T. D. FITCH, M.D.,
No. 296 W. Monroe St.
ERNST SCHMIDT, M.D.,
No. 385 State St.
WALTER HAY, M.D.,
No. 384 Michigan Ave., *Secretary.*

On the same evening, Oct. 20, Drs. Edgar and Hermann, the Committee appointed by the profession of St. Louis, arrived, bringing the first material aid to the sufferers, to the amount of ($900) nine hundred dollars, which was distributed by themselves, after consultation with the Committee, in eighteen checks of ($50) fifty dollars each, to an equal number of physicians who were deemed by the Committee, from the best information in their possession, to be in the most pressing want. These were placed on the following day, Oct. 21, in the hands of the Secretary (Dr. Hay) for distribution, the checks being drawn to the order of the beneficiaries respectively, and signed by the chairman of the St. Louis Committee.

On the 25th of October, the Committee were cheered by the receipt of the following letter:

No. 43 W. 32D ST., NEW YORK, Oct. 22d, '71.

*My Dear Doctor:*

We have received from the medical men and medical students of this city $4,700 and a little over, (and we hope this sum may be increased) in aid of the doctors who have suffered by fire in Chicago. I ought, however, to mention that the "Executive Committee" has authorized its chairman, Dr. Peaslee, and Dr. Hubbard, together constituting a "sub-Committee," to divert a portion of this money according to their discretion, to such physicians in other parts of the Northwest as have suffered from the recent fires. They also desire that, in disposing of the money sent to you from us, you will not overlook the claims of medical students who may have suffered in like manner. The medical students at our various medical colleges in New York have contributed very liberally to the common fund.

We are now waiting to hear from the medical men of your city, what organization they have effected, and who is authorized to receive the money.

As you, with other physicians of Chicago, have received communications both from the Pathological Society, and from a "Meeting of Physicians," of which Dr. Peaselee was chairman, it may be necessary to explain, that the action of the Pathological Society terminated, being merged into the later action of the meeting, and that both are represented by the "Executive Committee," in whose name and by whose direction I am now addressing you.

Will you be kind enough to lay these facts before the physicians of Chicago, and to request them to inform us who is authorized to receive the funds.

Very truly yours,

FRANK H. HAMILTON, M.D.,
*Chairman Executive Committee Chicago Doctors' Relief Fund Organization*, New York City.

JNO. H. RAUCH,

Board of Health, Chicago.

On the 24th of October, a communication was received from the Academy of Medicine, Cincinnati, through Dr. C. C. Comegys, its President, enclosing the sum of - - - - - - - - $392
Also, one from Dr. Francis Minot, of Boston, enclosing - 10
Also, from Dr. H. Kiefe, of Detroit, - - - - - - - 25
Also, from Dr. Mergler, of Wheeling, Cook Co., Ill., to be given to two of the poorest German physicians, - - - 20

By this date the number of applications for relief, sent in to the Committee, in compliance with its published notice, amounted to seventy-six. The Committee, upon consultation, decided to classify these applicants as follows:

1st. Those known, personally, or by satisfactory certificates, to be legitimate and honorable members of the profession, were placed upon the relief list;

2nd. Those unknown and not authenticated were placed upon the reserved list for investigation; and

3rd. Those known to be irregular, or, if regular, disreputable, were placed upon the rejected list.

The first class of this division was again subdivided into three, viz.:

1st. Men with families, who had lost residences, offices and practice, constituted the 1st class;

2nd. Those having lost only residences or offices, and had a portion of their practice left, formed a second class; and

3rd. Young men without dependents.

It was also decided to classify medical students in the same manner.

| | |
|---|---|
| There was at this date in the treasury, - - - - - | $447 |
| Which was apportioned thus : | |
| To three physicians of the first class, $50 each, - - | $150 |
| To five of the second class, $25 each, - - - - | 125 |
| And to nine of the third class, $10 each, - - - | 90 |
| To two German physicians $10 each, Dr. Mergler's donation, - | 20 |
| Stationery, printing and express, - - - | 5 |
| Leaving balance in treasurer's hands, - - - - | 57 |
| | $447 |

On Oct. 27 the following letter was received :

NEW YORK, Oct. 24, 1871.

DR. WALTER HAY—*Dear Sir :*

Enclosed you will please find my check for Two Thousand Dollars, payable to your order for the benefit of the suffering medical men of Chicago, of respectable standing in our profession. Please acknowledge the receipt of the same on reception.

(Signed) S. T. HUBBARD, M.D.,
*Treasurer*, No. 27 W. 9th St.

P. S. The circular on which was printed your transactions was misdirected, otherwise the check would have been sooner sent ; more will soon be sent you.

S. T. H.

On the 28th of October, further donations were received, as follows :

| | |
|---|---|
| From C. E. Buckingham, Boston, Mass., - - - - - - | $25 |
| From the Academy of Medicine, Cincinnati, O., through Dr. C. C. Comegys, President, - - - - - - - | 65 |
| From Franklin Bonney, M.D., Hadley, Mass., - - - - | 14 |
| | $104 |

At the meeting of the Committee on Oct. 28th, the number of applicants had increased to ninety-nine—of which seven had been rejected for the causes already decided upon by the Committee.

Of the ninety-two remaining, thirty-five had been already relieved as exhibited above, and to this number eighteen were added, making the total number of beneficiaries fifty-three.

| | |
|---|---|
| To six of these, seventy-five dollars each was awarded, - - | $450 |
| To one, sixty-five, - - - - - - - | 65 |
| To fifteen, fifty, - - - - - - | 750 |
| To twelve, thirty, - - - - - - - | 360 |
| To four, twenty-five, - - - - - | 100 |
| To eight, twenty, - - - - - - - | 160 |
| Total, - - - - - - | $1,885 |

| | | |
|---|---|---|
| Total receipts to date, | $3,451 | |
| Total disbursements, | | $3,175 |
| Balance in Treasurer's hands, | | 276 |
| | $3,451 | $3,451 |

November 1, a donation was received from the Kings Co. Medical Society of Brooklyn, per Dr. J. H. H. Burge, Prest., by Prof. Moses Gunn, and by him handed to the Committee, of $1,000. And on November 3d, the Secretary received from Dr. S. T. Hubbard, Treasurer of the New York Executive Committee, his check for $2,000.

On Saturday, Nov. 4, the Committee met to make its third apportionment, at which time the number of applicants had increased to one hundred and twelve. The total number of rejections sixteen. Twenty-one were suspended for further examination, and the beneficiary list was increased to seventy-five, of which—

| | | |
|---|---|---|
| One received | | $135 |
| Six received $100 each, | | 600 |
| Fifteen received $45 each, | | 675 |
| Ten received $35 each, | | 350 |
| Eight received $25 each, | | 200 |
| And nineteen received $15 each, | | 285 |
| Total, | | $2,245 |
| Amount received to date, | $6,588 | |
| " disbursed to date, | | 5,420 |
| Balance in hands of Treasurer, | | 1,168 |
| | $6,588 | $6,588 |

On November 6, a contribution was received from the German physicians of Baltimore, through Dr. A. Friedenwald, by Dr. Ernst Schmidt of this Committee, of $132, together with Dr. Friedenwald's personal contribution, $5. At the same date the following letter was received by Prof. Moses Gunn, and by him handed to the Committee:

17 CLINTON ST., BROOKLYN, NOV. 1, 1871.

PROF. M. GUNN—*Dear Doctor:*

Two of my colleagues in the Brooklyn Eye and Ear Hospital, Drs. Matthewson and Prout, and myself, are contributors to the fund raised by the Kings County Medical Society, for the profession in Chicago. Hearing that the

Chicago Eye and Ear Infirmary was destroyed by the fire, it would greatly please us if the sum we contributed, viz., ($75.00) seventy-five dollars, were placed in the hands of Dr. Edward L. Holmes to assist in the continuance of his clinic.

(Signed) H. G. NEWTON, M.D.

Dr. Newton has requested me to enclose this in the same envelope with my letter to you. It is not intended to be in any sense official. So far as the suggestion may seem to you and to your associates a wise one, it has my approval.

(Signed) J. H. HOBART BURGE, M.D.

Accompanying the above letter was a check for $100.

On the 23d of October, the following notice to the profession was issued in Philadelphia, of which copies were forwarded to the Committee :

PHILADELPHIA, Oct. 23, 1871.

*Dear Sir:*

The undersigned, a committee appointed by the Citizens' Executive Committee to aid the Chicago sufferers, earnestly request you to be present at a meeting of the profession, to be held at the Hall of the College of Physicians, N. E. Corner of Thirteenth and Locust Streets, on Wednesday, Oct. 25, at 8 P. M.

Respectfully, etc.,

(Signed) H. LENOX HODGE, M.D., *Chairman,*
THEO. A. DEMMI, M.D.,
WM. B. ATKINSON, M.D.,
R. M. GIRVIN, M.D.,
WM. H. PANCOAST, M.D.,
COMMITTEE.

WM. B. ATKINSON, M.D.,

*Secretary.*

The Committee have been informed, that at the meeting called by the above circular, it was decided to add the contributions of physicians to the General Relief Fund, raised by the citizens at large in aid of the Chicago sufferers.

The Committee regret this decision of our Philadelphia brethren by which the members of their own profession here, for whose aid their contributions were largely intended, will be cut off from its benefits.

| | | |
|---|---|---|
| Total receipts, up to November 15, - - - | $6,825 | |
| Disbursements to physicians, - - - | | $5,425 |
| " for incidentals, - - - - | | 10 |
| Balance in Treasurer's hands, - - - | | 1,390 |
| | $6,825 | $6,825 |

The fund has been distributed specifically, up to the present date, Nov. 15, as follows. Of those to whom relief has been given—

| | |
|---|---|
| One has received, | $165 |
| One has received, | 145 |
| Seven have received $135 each, | 935 |
| One has received, | 125 |
| Thirteen have received $100 each, | 1,300 |
| Four have received $90 each, | 360 |
| One has received, | 85 |
| One has received, | 80 |
| Five have received $75 each, | 375 |
| One has received, | 65 |
| Seven have received $50 each, | 350 |
| Thirty have received $45 each, | 1,350 |
| Three have received $30 each, | 90 |
| | $5,425 |

November 15, received from the New York Executive Committee, per Dr. S. T. Hubbard, Treasurer, $1,000, which leaves an unexpended balance in the Treasurer's hands of $2,405.

W. HAY, M.D.,
*Secretary.*

